# Conceptual Framework for Understanding Incident Management Systems During Public Health Emergencies

**DOI:** 10.1017/dmp.2022.77

**Published:** 2022-05-27

**Authors:** Aaron Clark-Ginsberg, Holly Fisher, Jalal Awan, Adriana Rico, Tracy Thomas, Dale Rose, Sara Vagi, Leecresia Jenkins, Christopher Nelson

**Affiliations:** 1 RAND Corporation, Santa Monica, CA, USA; 2 Centers for Disease Control and Prevention, Atlanta, GA, USA

**Keywords:** incident management, disaster response, performance evaluation, public health emergency preparedness, COVID-19 pandemic

## Abstract

**Objective:**

Effective incident management is essential for coordinating efforts of multiple disciplines and stakeholders when responding to emergencies, including public health disasters such as the ongoing coronavirus disease 2019 (COVID-19) pandemic.

**Methods:**

Existing research frameworks tend to focus on formal structures and doctrine (eg, ICS-NIMS); however, organizational processes that underlie incident management have not been systematically assessed and synthesized into a coherent conceptual framework.

**Results:**

The lack of a framework has hindered the development of measures of performance that could be used to further develop the evidence base and facilitate process improvement. To address this gap, we present a conceptual framework of incident management drawn from expert feedback and a review of literature on incident management and related fields. The framework features 23 measurement constructs grouped into 5 domains: (1) situational awareness and information sharing, (2) incident action and implementation planning, (3) resource management and mobilization, (4) coordination and collaboration, and (5) feedback and continuous quality improvement.

**Conclusions:**

As such, the article provides a first step toward the development of robust measures for assessing the performance and effectiveness of incident management systems.

Effective incident management is critical for coordinating response to emergencies, including public health disasters such as the ongoing coronavirus disease 2019 (COVID-19) pandemic.^1^ Incident management is the set of processes and activities in which risks are characterized, objectives are defined, and resources of different stakeholders are coordinated and deployed to address needs during an emergency situation.^2^ Among the primary outputs of effective incident management are answers to questions such as “Who is in charge?” “What resources are available?”, “How is the threat evolving?”, and “How will we pay for the response?”.^3^

Incident management, as a set of organizational processes and *activities*, is different than the *formalized structures* and *doctrine* that are typically part of a government’s disaster management policy. The most prominent incident management doctrine for the United States is the Incident Command System (ICS) and broader National Incident Management System (NIMS). Other governments also engage in incident management following their own doctrine: in Pakistan, incident management structures are in place for polio eradication; in Nigeria, malaria response is being managed through an incident management system; throughout West Africa, incident management systems were stood up during the 2013-2016 Ebola response; and globally as part of the ongoing COVID-19 response.^4,5^ International agencies also use incident management systems, such as the World Health Organization (WHO), whose Emergency Response Framework is designed to provide a common approach for the WHO’s response to emergencies.^6^

While public health agencies across the world use incident management systems as part of their response to health-related emergencies, it is unclear just how effective their systems are and how they can be improved.^7^ There are 2 reasons for this. The first is that incident management originated in the broader emergency management field outside of public health, and there is limited research on incident management for public health emergencies.^8^ Second, and more fundamentally, improving public health systems requires not just ascribing to formalized doctrine, but understanding of structure and process that could be linked to outcomes.^9^ And while many outcome-based health-care quality and patient safety metrics exist, measures for assessing structure and processes for public health emergencies, such as those related to incident management, are scant.^10^

A conceptual framework could be a useful first step toward developing measures of incident management system performance for public health incidents. Conceptual frameworks are representations of abstract, complex ideas. For researchers, they provide coherence for research design by informing research questions, methodologies, and data analysis,^11^ while they provide insight on what success might look like in practice for policy-makers and practitioners.

Therefore, the aim of this article is to develop a practical framework conceptualizing incident management systems that can be used as an initial basis for, ultimately improving how we measure performance and effectiveness of the management of public health-related incidents. Our research question is: how can we conceptualize incident management processes (apart from adherence to formal structures and doctrine such as ICS-NIMS) in a coherent framework that can be used as a basis for measuring the effectiveness of managing public health-related incidents? In what follows, we next describe the methods used to answer our question and develop this framework. We then describe the framework in detail, before concluding by discussing its practical implications and how it might be used to support future incident management research and evaluation, ultimately improving public health practice.

## Methods

Given that incident management includes a combination of explicit and tactical knowledge,^12^ we developed our framework in an iterative manner, leveraging existing academic literature and government policy to understand explicit knowledge and combining it with a series of expert elicitations to understand the tactical. We began by first developing an initial draft framework through a review and synthesis of incident management research, theory, and doctrine^8,13–15^ as well as organizationally focused research on how organizations operate reliably in complex and volatile task environments.^16–21^ We identified this literature through a snowball sample using key sources identified by the research team as a starting point, and a Boolean keyword search of English-language literature published since 2000 in Google Scholar, Web of Science, and PubMed (search terms were “public health” AND “incident management system” OR “incident command system”). We found 7870 nonduplicate articles through this process, which we narrowed down to 254 relevant articles through an abstract review. We then synthesized these articles to develop our initial framework, conceptualizing public health incident management as functionally comprised of 9 distinct domains, each with their own set of underlying constructs.

We then engaged in a series of expert elicitations to refine the framework. We started by having 11 local and state public health agencies across the United States rate the domains by importance and observability using a 5-point scale. From this and a set of associated discussions, we reduced our domains from 9 to 5. We then engaged in a series of hour-long expert discussions to further refine the framework and link it to the reality of incident management, revising the framework as feedback emerged from these discussions and sharing new versions of the framework until we reached saturation point, the point at which no new feedback was provided.

In total, we received feedback from 50 experts and went through 12 iterations of the framework, refining the domains and constructs as we went. The majority of the stakeholders we spoke with had expertise in emergency public health: 20 were experts from state and local health departments, and 6 from federal or national public health emergency preparedness agencies. State-level practitioners were recruited in consultation with the Association of State and Territorial Health Officials, whereas local-level practitioners were recruited in consultation with the National Association of County and City Health Officials. In addition, we consulted with 13 experienced responders from non-health disciplines but with experience in public health response and 5 academic researchers with expertise in incident management and incident management systems.

## Results

### Framework for Public Health Emergency Incident Management Systems

Our proposed framework is comprised of 23 constructs grouped into 5 domains (see Table 1). Each construct captures the activities that, based on the literature and our experts’ feedback, are critical to ensuring effective incident management for a variety of public health incidents, regardless of the specific incident management system used.

These domains, and their constituent constructs, are explained in detail below:

### Situational Awareness and Information Sharing

*Situational awareness and information sharing* refers to initial and ongoing perceptions and characterization of information from the environment to share information and identify ongoing response needs. Situational awareness is critical in assuring that response activities, including but not limited to the selection of medical and nonmedical countermeasures, are well matched to the threats confronting the public.

In Endsley’s foundational situational awareness framework, situational awareness involves 3 “levels”: (1) perception and intake of information; (2) sense-making, the ability to see the “big picture” by putting all the disjointed elements of information together, finding relationships between them, and assigning meaning to the scenario; and (3) projection, modeling, or the making of predictions about how the incident might unfold in the future.^22,23^ This involves routinely assessing information about threats (eg, in the context of infectious diseases, virulence, mode of transmission, severity) and their potential impact on at-risk and vulnerable populations, systems (eg, medication supply chains), and resource use (eg, medical countermeasures, deployed staff).

Following Endsley’s (1995) first “level,” construct 1 focuses on *determining what information is relevant*—and irrelevant. To be relevant, information must be credible, actionable, appropriate, timely, and strike the right balance between sharing too much and too little.^24^ Some experts described how staying on top of incoming information while synthesizing previous information could be highly challenging given the volume of information coming in coupled with the speed of incident response. This challenge can be particularly acute during an incident’s initial stages. This is due to a massive volume of information incident managers often encountered coupled with high levels of uncertainty about the incident and the necessary response strategies. Not surprisingly, many experts emphasized the importance of both the quantity and quality of information throughout the incident, noting that, without filtering, information could be a “firehose” that hinders rather than enhances response.

The complexity of many incidents means that there is often a wide range of potentially relevant information sources (eg, epidemiological data, reports from local health officials). Thus, construct 2 is *accessing and gathering information from multiple sources*. Similarly, sense-making in complex incidents requires group interaction to build sufficiently rich mental models that can help synthesize information and avoid the risk of missing the big picture,^25,26^ captured by construct 3 on *engaging in sense-making to maintain situational awareness*.

Because responding to public health incidents usually involves joint action by multiple partners, response leaders must share key information with appropriate partners and the public on an ongoing basis. The severity or duration of an incident can be affected if response leaders are unable to access, assess, document, and share relevant information, often across multiple jurisdictions and different levels of government.^27^ In addition, several experts noted the importance of *sharing information with external partners* (construct 4), including elected and appointed officials, impacted organizations, and other decision-makers, and *disseminating information to the public* (construct 5). Experts described *written documentation* (construct 6) as a critical tool to not only maintain continuity of response operations and support process improvement, but also provide evidence to resolve billing, litigation, and other investigatory activities (media requests, watchdog inquiries, etc.) that may occur. One government official responsible for county health-care incident management went so far as citing record keeping as one of the reasons for activating incident management systems, describing how activating incident management systems could be a way to help manage record keeping when it was beginning to present a challenge.

### Incident Action and Implementation Planning

*Incident action and implementation planning* involves the ability of incident managers to articulate and communicate decisions in coherent incident action plans in order to set up implementation of those plans. Decision-making is at the heart of incident action and implementation planning, since at the most basic level, decision-making refers to a commitment to action intended to yield desired results.^28^ Decision-making for complex incidents requires input from different departmental units, both in identifying and weighing pros and cons of various alternatives, and in minimizing disconnects between the decisions and execution^29,30^ (construct 7 is *identifying and assessing potential courses of action*). The experts we spoke with emphasized how they frequently needed to make decisions quickly and often with less-than-complete data to respond to rapidly evolving incidents.

Articulating clear decisions in an incident action plan (IAP) and handing those plans off to be implemented are also crucial parts of incident action and implementation planning. IAPs that successfully articulate clear decisions promote unity of effort and avoid situations in which responders receive conflicting messages. Thus, additional constructs are the *articulation of clear decisions* (construct 8) and the *development of an incident action plan* (construct 9).

The incident management team also needs to *hand off plans to implementers* (construct 10) including priorities, tactics, and timelines. Successful communication of these plans is critical for ensuring execution. Thus, situational awareness and decision-making compromise a feedback loop that promotes agility, adaptiveness, and recovery.^22,31^

### Resource Management and Mobilization

*Resource management and mobilization* involves identifying, accessing, deploying, and monitoring material, human, and organizational resources. Incident management depends on having the right resources at the right time, making resource management and mobilization a necessary part of effectively addressing public health threats.

In most incidents, determining when to stand up the formal incident management system is one of the first decisions that an incident management team needs to make (reflected in the first construct of this domain, *initiate stand up of IM system*, construct 11). Resource management and mobilization-related decision is closely tied to situational awareness, as it requires considering what is happening (Endsley’s (1995) perception), whether it is serious enough to warrant concern (characterization), and whether the situation is likely to improve without additional intervention (projection). As part of this process, decisions need to be made as to *the role public health might play in the incident* (construct 12). Regardless of the role of public health in the incident, the incident managers we spoke with described how late stand-up can put the response “behind the curve” at the outset; however, standing up the system too early can unnecessarily consume scarce resources from important routine public health activities.

Incident managers must also make *ongoing adjustments to the IM system structure* as needed (construct 13), including mobilizing and demobilizing staff, materials, and equipment. As with other decisions, resource management should reflect incident managers’ understanding of the situation, which may evolve as responders take actions to mitigate the incident or respond to external factors outside responders’ control. For instance, incident managers might change the partners that they engage with, the resources that they use, and shorten or elongate operational periods and *mobilize or demobilize staff and equipment* (construct 14), depending on incident changes.^8,32^

In all multijurisdictional responses, regardless of whether they are public health-related, resources are often “owned” or controlled by a wide variety of entities. Thus, incident managers described *resource tracking* (construct 15) as a particularly challenging part of incident management, sometimes to the point that they would create special processes solely to track resources. For instance, during the 2009 H1N1 response, the CDC IM team created a separate technical support unit for tracking resource requirements.^33,34^

Because public health incidents can potentially occur over an extended time period of weeks and months, as incidents wear on, incident management team members often face increasing pressure to attend to their routine duties.^35^ For extended incidents and others with time and resource constraints, incident managers described how they needed to navigate competing job requirements and be *cognizant of factors affecting team morale and fatigue* (construct 16), such as scheduling and stress.

Finally, *incident management systems must be demobilized* (construct 17) on an appropriate timeline to facilitate a smooth return to normal operations and enhance recovery. Information on proper demobilization is critical to ensure safety and a proper close to operations.^36^

### Coordination and Collaboration

Coordination and collaboration refers to the process of mutual adjustment between actors with views to reaching a common objective and maintaining unity of effort.^37,38^ The 2 concepts can be considered parts of a spectrum of cooperation: coordination being a basic level of cooperation—cooperating through sharing information—and collaboration a greater level of cooperation—actively working together to reach a shared objective.^39^ The result of good coordination and collaboration, therefore, is a plan in which the activities of various partners do not conflict and ideally are *mutually reinforcing* (construct 18).

*Establishing and maintaining roles, responsibilities, and legal authorities within IM system* (construct 19) by clarifying roles, goals and expectations of an incident is the first step in coordination and collaboration (eg, which agency has the lead response role and what are the limits of their authority). Because of the dispersed set of impacts incidents often have, these entities are often all necessary to manage an incident. Yet our experts described how in some cases it can be difficult to coordinate roles, responsibilities, and legal authorities within the IM system because differences between entities can lead to differences in priorities, which can result in conflicts over what to do.

Establishing and maintaining roles, responsibilities, and legal authorities within the IM system requires knowledge of the broader operating environment. It therefore depends on *awareness of IM system partner roles and responsibilities* (construct 20), yet differences between partners can make it difficult to build this awareness. *Using standard terminology* (construct 21) helps ensure participants can effectively communicate their roles and their priorities: for instance, 1 expert described how having a “common language,” allows new participants to join the response “without too much of a learning curve.” Documentation, communication, and clarification of legal authorities such as by establishing memoranda of understandings, was also cited as helping to maintain roles and responsibilities by institutionalizing roles and expectations. One expert noted the importance of informal “socialization” of incident management team members in fostering a mutually agreed and shared understanding during responses—something that can be difficult when use of incident management systems is infrequent, and in communities with high staff turnover.

### Feedback and Continuous Quality Improvement

*Feedback and continuous quality improvement* (CQI) refers to the practice of IM team members seeking, receiving, and using information about the outputs and outcomes of past operational periods to identify lessons for current and future responses. CQI may include capturing lessons about response activities and interventions, such as the reach and coverage of medical countermeasures administered, or risk communications disseminated. It may also include lessons about supporting processes and protocols, such as the procedures for how those medical countermeasures are administered or how information is shared with partners who disseminate communications. It should also include lessons from both within and across agencies involved in incident management, and between response partners and field personnel, particularly to document unplanned or unanticipated innovations that help the incident management team make progress toward objectives.

Feedback and CQI involves both *seeking and receiving feedback* (construct 22) and *using feedback to improve processes and outputs* (construct 23). The culture and values of an organization shape feedback and CQI: literature on high reliability organizations suggests that organizations effective in operating in complex task environments have cultures that emphasize and reward continuous learning.^40^ These learning cultures include “mindfulness” or a rich awareness of discriminatory detail, a capacity for action, preoccupation with failure, systems thinking, and incentive structures that reward reporting errors.^21,41,42^ Although organizations need learning cultures in order to maximize feedback and CQI, experts identified barriers impacting learning cultures. These include difficulty finding the time and resources to document lessons learned, and hesitation in surfacing weaknesses (for instance, to avoid political fallout and damage to relationships with response partners).

Feedback and CQI tends to occur after an incident is over, but efforts are being made to develop formal mechanisms for feedback and CQI during incidents. Post-incident feedback and CQI often takes the form of documentation of response-related strengths and weaknesses in an after-action report (AAR), a well-established practice in incident management and described in detail by the Homeland Security Exercise Evaluation Program.^43^ However, our experts noted that feedback and CQI are also important *during* an emergency to ensure response leaders can successfully adjust response operations as response priorities and circumstances change. Indeed, efforts are being made to improve CQI during emergency, such as through intra-action reporting mechanisms (see, eg, Chamberlin et al.^44^). By overcoming barriers impacting learning cultures and strengthening feedback and CQI during incidents, these mechanisms can help IM teams receive information and use it to enhance performance, ultimately improving their agility during response.

### Interactions Between Domains and Incident Management Context

While the proposed constructs are conceptually distinct, in reality they are operationally intertwined to some extent. For instance, once a public health threat has been identified and incident management processes have been established, the incident management team uses situational awareness and information sharing to make decisions and engage in incident action and implementation planning, mobilize resources, and coordinate and collaborate. These activities in turn guide when and how the incident management team shares information, maintains ongoing situational awareness, continually soliciting feedback and adjusting response priorities to ensure mission success. Ideally, these domains and constructs work in concert as part of a larger, complex system where incident management is more than the additive sum of its components but reflects the continuous interaction between components.

The specific manner in which these domains and constructs function depends on the complexity, novelty, spread, and severity of an incident. Many experts noted that most incidents start with a high level of uncertainty (eg, due to unknown origin or scale of public health threat) before response leadership begins the process of making sense of the incident. Bigley and Roberts^16^ similarly describe how incidents become less complex as the incident management team establishes structures that put a pattern around the incident. Likewise, interconnections between domains may also change over the course of an incident. For instance, initial situational awareness may both shape and be shaped by resource management and mobilization during large scale ups or scale downs.

## Discussion

Existing lite0rature on incident management is scattered and has provided few, if any, integrated conceptual frameworks of IM processes (apart from adherence to formal structures and doctrine such as ICS-NIMS) that can be used to understand the key tenants of incident management and further develop the evidence base and support system improvement efforts on the front lines. This article has developed such a framework, in the form of 23 constructs organized into 5 domains, that can be used as a basis for measuring performance and effectiveness and to support improving the management of public health-related incidents. The framework highlights that incident management begins with both sense-making and decision-making, which ideally occurs even outside the context of incident management. Once initiated, numerous other processes—from information sharing, to coordination, role identification, resource management, and staff continuity and support—are harnessed to improve situational awareness and to support action planning, guided by sense-making and decision-making.

The proposed framework focuses on the actual activities and processes associated with incident management but does not include constructs related to developing and sustaining the ability to execute these incident management activities. These include input elements such as resources (eg, surveillance systems) and contextual factors like organizational structures (eg, ICS-NIMS), and culture (eg, trust, willingness to share information), the latter of which is emphasized in literature on high reliability organizations.^16,21,45^

Our framework is designed to be used as a basis for developing practical measures of incident management, which can lead to greater understanding of incident management in health contexts and improved performance and effectiveness in managing real-world incidents. Currently, we are conducting feasibility testing of draft incident management measures based on this framework at various US and international government agencies supporting the COVID-19 response. These measures are being designed to be used to assess the effectiveness of incident management systems for public health emergencies, including COVID-19 as well as a range of other incident types, which to date has been limited by the absence of a comprehensive evidence-informed framework. Developing high-quality measures for public health emergencies will help support continued development of an evidence base for evaluating response processes, ultimately improving public health.

Our framework may also have utility for non-public health related incidents and for incident management in other countries. As our experts noted, non-public health related emergencies are also complex, fluid, and highly uncertain, and thus require incident management strategies similar to health incidents. Additionally, although our framework is based on discussions with US emergency managers, approaches to public health incident management are increasingly universal as a result of factors such as exchanges and cross-country learnings across countries and increasingly standardized incident management expertise.^46^ The framework we develop is designed to capture these processes in a way that is agnostic to the doctrine or policy of the organization managing the incident. While we expect our framework to be useful for public health incident management across a variety of response contexts, future research should explore more systematically its utility in different contexts and settings given potential differences in incident management effectiveness.

## Conclusions

This article provides a conceptual framework of incident management systems for public health emergencies that can be used as a basis for developing measures of performance and effectiveness of the management of public health-related incidents. This comprehensive framework is founded on the scientific literature, existing practices at governmental and nonprofit agencies, and the expertise of 50 emergency management professionals. Public health incident management is a complex process that requires emergent and adaptive management to changing incident contexts in a structured and formalized way. The framework presented in this article provides a steppingstone for researchers, practitioners, and policymakers in various health and nonhealth emergency response organizations to view, assess, and improve performance of their respective organizations in dealing with common and novel health-related emergencies.

## Figures and Tables

**Table 1.** Conceptual framework for understanding Incident management systems during public health emergencies

	Domain	Relevant Constructs	SME Feedback on Domain/Construct(s)
1	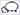 **Situational Awareness and Information Sharing:** Initial and ongoing perception and characterization of information from the environment to share information and identify ongoing response needs	1. Determining what information is relevant 2. Access and gather information from multiple sources 3. Engage in sensemaking to maintain situational awareness 4. Information sharing among appropriate partners (internal and external) 5. Disseminate information to public 6. Documentation	*“. information gets watered down (as you go) further down the chain of command, making it harder to figure out what the incident commander really wants”* *“Confusion creates chaos”* *“. crucial to figure respondents with unmet data needs, as seen in the 2014 Ebola response”* -
2	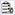 **Incident Action and Implementation Planning:** Incident managers articulate and communicate decisions in coherent incident action plans to set up implementation of those plans	7. Identify and assess potential courses of action 8. Articulate clear decisions 9. Develop Incident Action Plan 10. Hand off Incident Action Plan to be implemented	*“All levels of response should know what is happening and why?”* *“Second-hand or verbal knowledge may not be as reliable”*
3	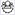 **Resource Management and Mobilization:** In consultation with appropriate stakeholders across agencies, incident managers deploy human, physical, and other resources in a manner that matches ongoing situational awareness, identification of roles, and relevant decisions	11. Initial stand up of IM system 12. Ongoing and timely determination of public health role 13. Ongoing adjustments to IM system structure, as needed 14. Ongoing mobilization/demobilization of staff, materials, and equipment 15. Resource tracking 16. Monitor and manage staff safety, fatigue, and well-being 17. Final demobilization of IM system	*“… late stand-up can put the response “behind the curve” at the outset”* *“Shift length is unhelpful (to determine stress, fatigue), team morale is key”*
4	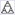 **Coordination and Collaboration:** Process of mutual adjustment between actors involved in incident management to reach a common objective	18. Mutually reinforcing activities in plans 19. Establish and maintain roles, responsibilities, and legal authorities 20. Awareness of partner activities 21. Use of standard terminology	“Shared experience, a clear chain of command and (common) language is key, especially in multi-jurisdictional incidents.” *“. important to leverage leadership to break logjam in cross-jurisdictional info sharing”* *“. nationally standardized but not federalized language helps”* *“Other state personnel need to be able to fit into emergency management when they are in town.”*
5	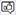 **Feedback and Continuous Quality Improvement:** Incident management staff seek, receive, and use information about outputs/outcomes of past operational periods to identify lessons for current and future responses	22. Seek and receive feedback 23. Use feedback to improve processes and outputs	*“Ongoing learning and adaptation is more important than formal (retrospective) reviews”*

*Note:* The framework consists of 23 constructs groups into five domains that together comprise the key elements of public health incident management. Each construct represents a distinct process that is critical for managing public health emergencies. Constructs and domains were developed from literature review and iterative discussions with 50 subject matter experts.
